# Pediatric radiation enteritis with intestinal failure: A case report and literature review

**DOI:** 10.1097/MD.0000000000020905

**Published:** 2020-06-19

**Authors:** Luojia Xu, Youyou Luo, Jindan Yu, Jingan Lou, Xiaofei Chen, Jie Chen

**Affiliations:** Department of Gastroenterology, Children's Hospital, Zhejiang University School of Medicine, National Clinical Research Center for Child Health, Hangzhou, Zhejiang Province, China.

**Keywords:** enteral nutrition, nutrition therapy, parenteral nutrition, radiation injuries

## Abstract

**Rationale:**

Chronic radiation enteritis, a disease secondary to radiation exposure, has been widely reported in adults. However, few studies have described chronic radiation enteritis in children. Early diagnosis is essential, and nutrition management plays an important role in pediatric chronic radiation enteritis management.

**Patient concerns:**

A Chinese 3-year-10-month-old boy was admitted with vomiting, weight loss (1–2 kg) after radiotherapy for a neuroblastoma.

**Diagnoses:**

The patient was diagnosed as neuroblastoma (primary site: left adrenal grand; site of metastasis: multiple bone metastasis, bone marrow invasion, intraperitoneal lymph node metastasis) in 2015. Five months after radiotherapy, he showed vomiting and weight loss with stricture in intestine and thickening intestinal wall in imaging finding. His daily intake was not sufficient and extra supplements were needed by intravenous infusion. He had a weight-for-age *z* score of −5.04, a weight-for-height z score of −6.19, a height-for-age *z* score of −2.22, and a body mass index-for-age *z* score of −5.87. The highest level of alanine aminotransferase was 1433 U/L. Those findings established a diagnosis of chronic radiation enteritis with intestinal failure, intestinal stenosis, severe malnutrition, and hepatic dysfunction.

**Interventions:**

This patient was treated by parenteral nutrition with minimal enteral feeding. Other treatments were aiming at complications during hospitalization.

**Outcomes:**

The patient weaned off parenteral nutrition finally with nutrition status and quality of life improved. There were no signs of tumor recurrence during the 4-year follow-up.

**Lessons:**

Pediatric radiation enteritis is rare. Our study highlights the characteristics of pediatric chronic radiation enteritis. Nutrition therapy is an important part of the whole therapy strategy in pediatric chronic radiation enteritis.

## 1 Introduction

Cancer therapy prolongs the patients’ survival time, but the toxicity related to therapy should be considered. Radiotherapy is one of the most effective treatments in cancer therapy and plays a central role in 25% of all cancer treatments.^[1]^ Radiation enteritis is a common complication of radiotherapy. Radiation enteritis can be subdivided into acute and chronic forms. Acute radiation enteritis occurs when radiation directly damages the intestinal mucosa and can usually spontaneously resolve over 2 to 12 weeks.^[2]^ In contrast, chronic radiation enteritis (CRE) develops months to years after radiation exposure^[2]^; the symptoms of CRE can vary from case to case.^[3]^ Due to radiotherapy being widely used in cancer therapy, the incidence of CRE has also become high.^[4]^ CRE occurs in up to 50% of adult patients^[5]^ and is associated with intestinal failure.^[3,6]^ Despite the advance of technology of radiation therapy, the mortality of CRE did not decrease.^[5,7]^ However, there are only a few studies on pediatric radiation enteritis. Here, we report a pediatric patient diagnosed as CRE with intestinal failure, and our aim is to expand the knowledge and experience of management pediatric CRE cases.

## 2 Case presentation

### 2.1 Case report

A 3-year-10-month-old Chinese boy was admitted to our department with vomiting, weight loss (1–2 kg) over the last 10 months. He started vomiting 10 months ago and his liver enzyme levels deteriorated to a maximum alanine aminotransferase (ALT) level of 1433 U/L. He received radiotherapy 5 months before the symptoms occurred. No fever, jaundice, abdominal distension, or diarrhea was noted. He irregularly received parenteral nutrition and nasogastric feeding with special formulas. Nevertheless, he lost 1 to 2 kg over the course of the illness. He decreased his daily activities because of the resulting weakness.

The patient was diagnosed as neuroblastoma (primary site: left adrenal grand; site of metastasis: multiple bone metastasis, bone marrow invasion, intraperitoneal lymph node metastasis) 22 months ago, his N-myc was amplified without 1p and 11q loss of heterozygosity (LOH), and he was classified as children's oncology group (COG) high risk. He was treated with chemotherapy, surgery (primary tumor resection), autologous peripheral blood stem cell transplantation, and participated in a clinical trial with an anti-disaloganglioside(GD2) antibody. Fifteen months before admission, the patient received radiotherapy for the primary site with a total radiation dose of 25.2 Gy and frequency of 14 times. His personal and family history was unremarkable.

### 2.2 Physical examination

He showed stable vital signs, and his body weight was 8.10 kg with a height of 93.0 cm, which indicated a weight-for-age *z* score (WAZ) of −5.04, a weight-for-height *z* score (WHZ) of −6.19, a height-for-age *z* score (HAZ) of −2.22, anda body mass index-for-age *z* score (BAZ) of −5.87 (calculated by the WHO Anthro version 3.2.2 software). Both lungs were clear, and cardiac auscultation was normal. The abdomen was soft with a 10 cm scar in the middle. There were signs of masses or hepatosplenomegaly during abdominal palpation.

### 2.3 Laboratory examinations

The liver function test showed that ALT ranged from 31 to 290 U/L and aspartate aminotransferase ranged from 47 to 174 U/L, whereas bilirubin, globulin, and albumin were in the normal range. Urine analysis and routine stool analysis results were normal. Other laboratory studies revealed a normal complete blood count and kidney and pancreas function. Blood lipids, folic acid, vitamin B12, ferritin, trace elements, vitamin D, vanillylmandelic acid, GD2, and neuron-specific enolase were in the normal range.

### 2.4 Imaging examinations

**Figure 1:**
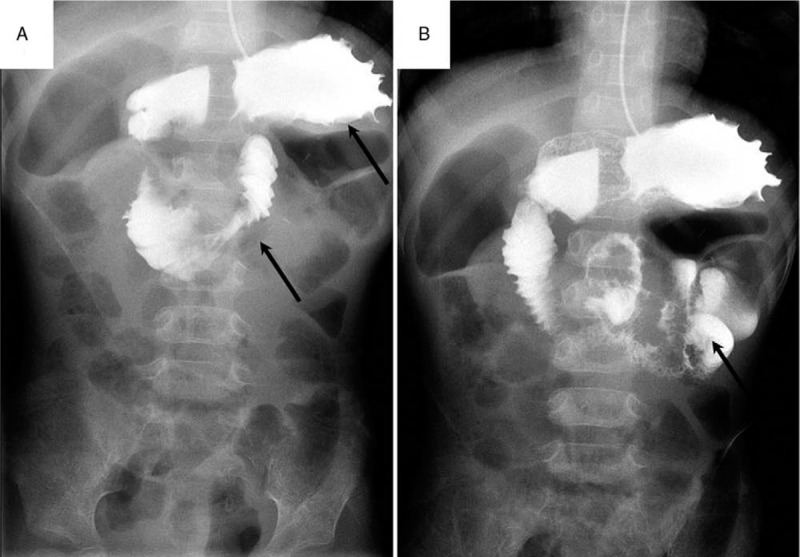
Gastrointestinal barium meal radiography demonstrates an unsmooth outline of fundus of stomach, a stricture between the duodenum and proximal jejunum (arrow, A), and featheriness or spring-like structure cannot be observed in jejunum (arrow, B).

**Figure 2:**
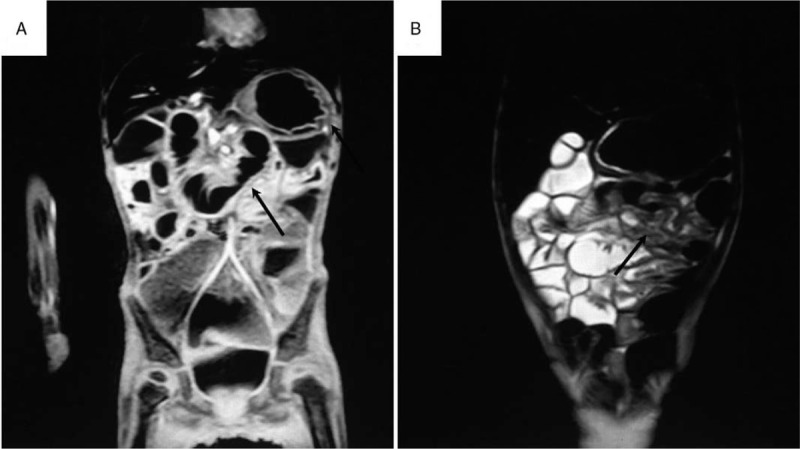
Coronal post-contrast spectral presaturation with inversion recovery T1-weighted magnetic resonance (MR) image demonstrates a stricture between the duodenum and proximal jejunum, thickening of gastric wall (arrow, A). Coronal pre-contrast T2-weighted MR image demonstrates multisegmental intestinal wall thickening and intestinal canal collapsed in mid-upper abdomen (arrow, B).

Gastrointestinal barium meal radiography demonstrated an unsmooth outline of the fundus of the stomach, a stricture between the duodenum and proximal jejunum (Fig. 1A and B), delayed gastric emptying, and inflation and dilation of the colon. Magnetic resonance enterography (MRE) showed strictures in the same place, thickening of the gastric wall and multisegmental intestinal wall in the mid-upper abdomen, enhanced after gadolinium injection, and no obvious mass in the left adrenal region (Fig. 2A and B). Esophagogastroduodenoscopy (EGD) with biopsy showed nonspecific changes but chronic inflammation in mucosa.

### 2.5 Final diagnosis

CRE (Grade 1),^[8]^ intestinal failure, intestinal stenosis, severe malnutrition, hepatic dysfunction, and postoperative of neuroblastoma.

### 2.6 Treatment

The main aim of treatment was to improve his nutritional status and quality of life without tumor recurrence. Enteral nutrition tried firstly, but he showed intolerance with vomiting on different ways of feeding ways or formula. Partial parenteral nutrition (PPN) was applied to meet his caloric and nutrient intake needs. Because his peripheral veins were limited as a result of a catheter-related infection and thrombosis, PPN could only provide limited nutritional needs. Then, the treatment plan changed to the following: after anti-infective and thrombolytic therapy, a peripherally inserted central catheter was placed along with antibiotic lock therapy (vancomycin 2 g/L). Then, parenteral nutrition became the main part of nutrition therapy and could provide an energy intake of 242.8 to 356.8 kJ/(kg·d), with a medium long-chain fat emulsion 1.0–1.5 g/(kg·d), ω-3 fish-oil lipid emulsion 1.0 g/(kg·d), amino acids 1.5–3.0 g/(kg·d), and glucose 7.9 to 12.0 g/(kg·d). Meanwhile, minimal enteral feeding (MEF)^[9]^ was applied with formula at a dose of <0.02 L/(kg·d) with an energy of <83.6 kJ/(kg·d). The patient's nutritional status improved, and he could tolerate increasing enteral nutrition. Therefore, we decreased the PPN dose and increased enteral nutrition. Finally, the patient could be weaned off PPN and tolerated an oral intake of 0.8 to 1.0 L formula per day, which provided an energy of 247.9 to 309.8 kcal/(kg·d). In total, parenteral nutrition was provided for 227 days, including 12 days on total parenteral nutrition, and MEF was provided for 50 days. During nutrition therapy, we weekly monitored the hepatic function, renal function, electrolytes, trace nutrients, serum lipids, and so on. Complications during therapy, such as catheter-related sepsis, thrombosis, deteriorated liver enzyme levels, and diarrhea, could be treated.

### 2.7 Outcome and follow-up

**Figure 3:**
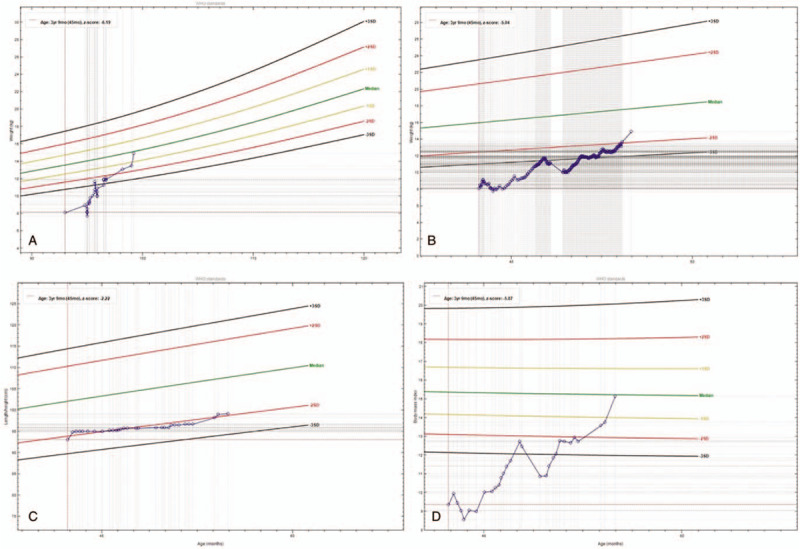
The patient's growth curve during nutrition therapy: weight-for-height *z* score (WHZ) (A), weight-for-age *z* score (WAZ) (B), height-for-age *z* score (HAZ) (C), and body mass index-for-age *z* score (BAZ) (D) demonstrate a catch-up growth during nutrition therapy. Green lines mean the median level. Yellow lines mean 1 standard deviation (SD) and red lines mean 2SD. Black lines mean 3SD. *z* score is negative when below green lines and is positive when above green lines.

When the whole therapy regimen finished, the body weight of the patient was 14.09 kg, and his height was 99.2 cm. Other anthropometric results are shown in Figure 3. The results of the serum tumor marker test, ultrasounds of abdominal organs, and ^123^I-labeled metaiodobenzylguanidine scan revealed no signs of tumor recurrence. During the 4-year follow up, the patient maintained his tolerance of enteral nutrition by oral intake with volume 0.4 to 0.5 L daily. Vomiting occasionally occurred but was not as severe as previous episodes. His quality of life also improved by regaining daily activities. When the amount of daily intake decreased, he intermittently needed readmission for a couple of days for PPN.

## 3 Discussion

There is little information about CRE in children. We have summarized the relevant literature from the past 40 years by searching the PubMed and Chinese databases with the key words “radiation enteritis” and “children.” There are 4 articles^[10–13]^ that included 18 patients with pediatric CRE (Table 1). The symptoms, management strategies, and outcomes are shown in Table 1.

**Table 1:**
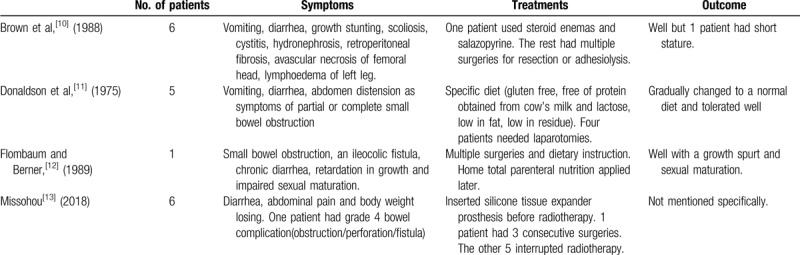
Summary of symptoms and treatments in pediatric chronic radiation enteritis.

### 3.1 Incidence and clinical manifestations

The occurrence of radiation enteritis is related to radiation dose, fractionation schedule, and the combination with chemotherapy.^[13]^ The incidence of CRE in adults is approximately 20% of all patients who received pelvic radiotherapy in the past 2 decades.^[14]^ The incidence in children remains unknown, but is probably very low. The case presented in this report reveals a pediatric patient with CRE who had main symptoms of vomiting and weight loss 5 months after radiotherapy, which is consistent with previous studies.^[2,3]^ In addition to radiotherapy, the patient reported here also received chemotherapy. Radiotherapy in this case only targeted the left adrenal grand, and radiation damage to the colon or rectum might be less severe than in other cases with pelvic tumors. This could explain why diarrhea was not as obvious here but was common in other studies.^[10–13]^

### 3.2 Imaging findings

To date, there are few pediatric imaging findings of CRE using MRE.^[15]^ The MRE findings for CRE are nonspecific, including wall thickening, luminal narrowing, and intestinal dilation. However, information from MRE can differentiate CRE from tumor recurrence and is helpful for evaluating the whole intestinal wall. In addition, MRE also presents all the possible lesion sites of the intestine at the same time, which is important for treatment planning, and especially, for making surgery decisions. The patient reported here underwent MRE, which showed a stricture in the small intestine and several segments of thickened intestinal walls. These characteristics are consistent with the MRE findings in adult studies.^[15]^ Our patient also had thickening of the gastric wall on MRE, which has not yet been reported, and this might indicate that the stomach also incurred radiation damage, which contributed to the recurrent vomiting. Thus, because of its advantage^[16–18]^ of no radiation exposure, MRE may be a suitable way to evaluate pediatric CRE patients.

### 3.3 Treatment

Malnutrition is a common issue in CRE because of the deteriorated intestinal functions of digestion, absorption and motility. Malnutrition in cancer patients increases mortality.^[19]^ Because pediatric patients have unique nutrition requirements for growth and development, optimal nutrition support in pediatric CRE patients is much more important than in adults. In children, malnutrition is associated with defects in host defenses and delayed puberty.^[12]^ In the case here, parenteral nutrition was vital. Meanwhile, MEF was applied during therapy to maintain the physiological rhythm of the intestine. There are several studies^[20–23]^ about MEF that focus on preterm feeding, but not many studies focus on elder children. A study^[24]^ on animal models suggested that MEF accelerated recovery after methotrexate-induced gastrointestinal mucositis. Here, our case showed that appropriate nutrition therapy, including a combination of MEF and parenteral nutrition for several weeks, is effective in improving nutritional status, intestinal rehabilitation, and growth without parenteral nutrition-associated cholestasis. The challenging parts of this therapy are as follows: at the beginning, the patient suffered from a malnutrition-infection cycle with thrombosis, which made it difficult to obtain a central venous line; his intestinal failure with poor nutritional status made enteral nutrition impossible at first, which suggested parenteral nutrition might be the first choice for his condition; hepatic dysfunction with a maximum ALT of 1433 U/L also limited nutrition therapy. Our experience in this case demonstrated that as soon as the thrombosis and infection are cured, nutrition therapy should be put into practice. Antibiotic lock therapy has been shown to be a good way to reduce the chance of catheter-related infection.^[25]^ We monitored hepatic function weekly, or more frequently when an infection occurred. If ALT was elevated, we changed the kind of lipid emulsions (changed medium long-chain fat emulsion to ω-3 fish-oil lipid emulsion) or reduced the composition of the parenteral nutrition dose (fat and amino acids).

Surgery is another option to treat CRE. However, CRE is a progressive disease that leads to poor regeneration capacity in tissue that has been damaged by radiation,^[26]^ and not all CRE patients require surgery. According to a study from France,^[27]^ approximately one-third of adult CRE patients undergo surgeries. The data of pediatric CRE patients^[10–13]^ show that 8^[10,12,13]^ of 18 patients had surgeries during the disease course. CRE patients with strictures,^[28]^ fistulas, perforations, and obstructions^[26]^ should be treated surgically. There were no signs of perforation or fistula in the case presented here. The patient had >1 lesion segment in the gastrointestinal tract, and his symptoms of vomiting and abdominal distension could be minimized by reducing the enteral nutrition dose or fasting for a few days. He did not require surgeries during the disease course.

## 4 Conclusion

Pediatric CRE remains rare according to the published literature. More experience about how to diagnose and manage this condition will be needed. This case report discusses the clinical features of pediatric CRE with intestinal failure and highlights the importance of nutrition management.

## Author contributions

**Conceptualization:** Luojia Xu, Youyou Luo, Jindan Yu, Jingan Lou, Jie Chen.

**Data curation:** Luojia Xu, Xiaofei Chen.

**Formal analysis:** Luojia Xu.

**Investigation:** Youyou Luo, Jindan Yu, Jingan Lou.

**Methodology:** Jie Chen.

**Supervision:** Jie Chen.

**Writing – original draft:** Luojia Xu.

**Writing – review & editing:** Youyou Luo, Jie Chen.
